# Movement behaviours, breakfast consumption, and fruit and vegetable intake among adolescents

**DOI:** 10.1186/s44167-022-00001-5

**Published:** 2022-10-01

**Authors:** Hugues Sampasa-Kanyinga, Hayley A. Hamilton, Jean-Philippe Chaput

**Affiliations:** 1 Healthy Active Living and Obesity Research Group, Children’s Hospital of Eastern Ontario Research Institute, 401 Smyth Road, Ottawa, ON K1H 8L1 Canada; 2 School of Epidemiology and Public Health, University of Ottawa, Ottawa, ON Canada; 3 Centre for Addiction and Mental Health, Institute for Mental Health Policy Research, Toronto, ON Canada; 4 Dalla Lana School of Public Health, University of Toronto, Toronto, ON Canada

**Keywords:** Physical activity, Screen time, Sleep, Breakfast, Fruits, Vegetables, Youth

## Abstract

**Background:**

It is recommended that children and adolescents spend ≥ 60 min per day of moderate-to-vigorous physical activity, ≤ 2 h per day of recreational screen time, and 9–11 h of sleep per night for school-aged children or 8–10 h per night for adolescents. The objective of this study was to examine the associations of compliance with physical activity, screen time, and sleep duration recommendations with the frequencies of breakfast consumption and fruit and vegetable intake among adolescents.

**Methods:**

Data from a cross-sectional and province-wide survey of students in grades 7–12 in Ontario (Canada) were used for this analysis (n = 12,759 students; 15.2 ± 1.8 years; 56% females). Movement behaviours and eating habits were self-reported. Multivariable ordered logistic regression analyses were adjusted for age, sex, ethnoracial background, subjective socioeconomic status, and body mass index z-score.

**Results:**

Compliance with all three recommendations was associated with more frequent breakfast consumption (OR: 2.77; 95% CI: 2.17–3.55) and fruit and vegetable intake (OR: 2.95; 95% CI: 2.41–3.62) compared with compliance with none of the recommendations. Compliance with the different combinations of recommendations was also associated with more frequent breakfast consumption and fruit and vegetable intake, with some exceptions. There was a dose–response gradient between the number of recommendations met (3 > 2 > 1) and more frequent breakfast consumption (p < 0.001) and fruit and vegetable intake (p < 0.001), with compliance with all three recommendations being the best combination.

**Conclusions:**

These findings suggest that compliance with the physical activity, screen time, and sleep duration recommendations is associated with more frequent breakfast consumption and fruit and vegetable intake among adolescents.

## Introduction

Adolescence is the transitional stage from childhood to adulthood that occurs between ages 13 and 19. It is a time of significant physical, emotional, psychological, social, and mental changes and growth [1, 2]. Nutritional needs during adolescence are increased because of the rapid growth and changes in body composition associated with puberty [3]. However, research has shown that healthy eating behaviours become less common during adolescence, with a decrease in the frequency of breakfast consumption and fruit and vegetable intake [4, 5]. While not being offered breakfast and fruits and vegetables may be a reason for not eating them in younger children, a body of research has reported a variety of other reasons amongst adolescents, such as lack of time in the morning, not feeling hungry, and/or for weight control [6, 7].

Breakfast has been identified as the most important meal of the day, and its regular consumption has been associated with a wide range of positive health and cognitive outcomes [8, 9]. The American Academy of Pediatrics recommends that children and adolescents consume breakfast for healthier body weights, improved nutrition, better memory, better test scores, and better attention spans [10]. Similarly, the consumption of fruits and vegetables is a primary component of a healthy dietary pattern [11]. It provides an essential source of vitamins, minerals, and fiber, and has been indicated to reduce the risk of heart diseases, digestive problems, and some types of cancer [11, 12]. Healthy eating behaviour, such as adherence to the Mediterranean diet which is characterised by high consumption of fruits and vegetables and other healthy components (such as bread, legumes, nuts and seeds, a low intake of red meat, a low-to-moderate consumption of wine, fish and poultry, and the use of olive oil as the principal source of fat [13]) has also been associated with positive academic performance among adolescents [14–16]. Identifying factors that could foster healthy eating behaviours among adolescents is necessary because the latter contributes to maintaining a healthy weight, promotes health, optimizes growth, and improves physical and intellectual development across the lifespan.

Research has shown that healthy movement behaviours, such as active living and better sleep, are associated with healthy eating habits among adolescents [17, 18]. Likewise, unhealthy lifestyle behaviours are associated with unhealthy eating habits [18, 19]. For example, Zakrzewski-Fruer et al. [20] found that frequent breakfast consumption was associated with higher physical activity time and lower sedentary time in the morning when compared with rare breakfast consumption in a multinational sample of children aged 9–11 years. Silva and Silva [21] found that low levels of physical activity were associated with inadequate fruit and vegetable intake in a sample of more than 2000 adolescents aged 13 to 18 years in Brazil. In a systematic review of 53 studies, Pearson and Biddle [22] found that sedentary behaviour, usually assessed as screen time and predominantly TV viewing, was associated with unhealthy dietary behaviours in children, adolescents, and adults, including lower fruit and vegetable consumption; higher consumption of energy-dense snacks, drinks, and fast foods; and higher total energy intake. With respect to sleep duration, Bel et al. [23] found that short sleep duration was associated with lower dietary quality in a sample of more than 1500 European adolescents.

An important limitation of the available evidence in this field of investigation is that movement behaviours (i.e., physical activity, sedentary behaviour, sleep duration) have been mainly examined individually and in isolation of each other in relation to dietary behaviours. The Canadian 24-h movement guidelines for children and adolescents represent a new movement paradigm that emphasizes the integration of all movement behaviours occurring over a whole day, shifting the focus from the individual components to the whole [24]. These guidelines recommend that children and adolescents accumulate at least 60 min of moderate-to-vigorous physical activity per day, limit their recreational screen time to no more than two hours per day, and have an uninterrupted 9 to 11 h of sleep per night for children aged 5 to 13 years or 8 to 10 h of sleep per night for adolescents aged 14 to 17 years [24]. In a study of more than 5800 children aged 9 to 11 years from 12 countries around the world, Thivel et al. [25] found that compliance with the 24-h movement guidelines was associated with better dietary patterns using a composite score. However, the association between compliance with physical activity, screen time, and sleep duration recommendations with specific eating behaviour indicators among adolescents, such as frequency of breakfast consumption and/or fruit and vegetable intake, is largely unknown. Investigating the relationship between compliance with physical activity, screen time, and sleep duration recommendations with eating behaviours among adolescents is crucial because the latter can directly influence adolescent development and well-being. More importantly, whether the combination of movement behaviours is most strongly associated with eating behaviours compared to adhering to single movement behaviours can help provide further support to the Canadian 24-h movement guidelines.

Therefore, this study aims to examine the association between compliance with combinations of the current recommendations for physical activity, recreational screen time, and sleep duration with consumption frequency of breakfast and fruits and vegetables among adolescents in Ontario, Canada. We hypothesized that adherence to the physical activity, screen time, and sleep duration recommendations would be associated with more frequent breakfast consumption and fruit and vegetable intake.

## Methods

### Sample and design

Data were derived from the 2019 cycle of the Ontario Student Drug Use and Health Survey (OSDUHS), a repeated cross-sectional survey of students in grades 7 through 12 in publicly funded schools in Ontario, Canada [26]. OSDUHS has been administered biennially in a random sample of Ontario schools since 1977. This research was performed in accordance with the Declaration of Helsinki, and ethics approval was obtained from the Research Ethics Boards of the Centre for Addiction and Mental Health and York University, as well as 31 school board research review committees. All students gave their signed assent in addition to parentally signed consent for those aged under 18 years before they participated in the study. The study design and methods are described in greater detail elsewhere [26].

The survey uses a stratified two-stage cluster design (school, class) and involved students from 47 school boards, 263 schools, and 992 classrooms in the 2019 cycle. A total of 14,142 students aged 11 to 20 years completed an anonymous self-administered pen and paper questionnaire in class [26]. The student participation rate was 59%, which is considered above average for a student survey that required active consent from a parent or guardian [27]. Students were lost due to absenteeism (12%), and to either unreturned consent forms or parental refusal (29%). Participants were excluded from current analyses if they were missing information on any of the variables included in the present study. This left a final analytic sample of 12,759 students. Participants with missing data were more likely to be males, in lower grades, and of White and Black ethnoracial backgrounds. Proportions, medians, and means were used to describe the data.

## Measures

### Dependent variables

Frequency of consumption of breakfast and fruits and vegetables was measured using items that were adapted from the Centers for Disease Control's (CDC) Youth Risk Behavior Surveillance System (YRBSS) [28] and the World Health Organization's Health Behaviour in School-aged Children (HBSC) study [29]. These international surveys have demonstrated a high test–retest reliability amongst middle and high school students [30–32]. Their breakfast consumption and fruit and vegetables intake measures have been widely used in research [4, 33, 34].

Breakfast consumption was measured using a question that asked participants to indicate the number of days they ate breakfast over the past five school days. Response options ranged from 1 (none) to 4 (all five days) and were treated as an ordinal variable, such that higher scores indicate more frequent consumption of breakfast.

Fruit and vegetable consumption was measured using a question that asked participants to indicate the number of times they eat fruits and vegetables on an average day. Response options ranged from 1 (0 times a day) to 7 (6 or more times a day) and were treated as an ordinal variable, so that higher scores indicate more frequent consumption of fruits and vegetables.

### Independent variables

Physical activity was measured using a question that asked participants to indicate on how many days they were physically active for a daily total of at least 60 min over the past 7 days. All durations spent in any types of physical activity that increased heart rate and made breathing hard some of the time were captured. Some examples of physical activity were provided, including running, brisk walking, biking, rollerblading, skateboarding, dancing, swimming, football, basketball, and soccer. Participants were also asked to include both activities that happened at school and outside of school. Response options ranged from 0 to 7 days. For analysis purpose, students who indicated “7 days” were considered compliant with the physical activity recommendation [24]. Those who indicated less than 7 days were considered non-compliant with the physical activity recommendation. Using a sample of 123 Australian adolescents from three secondary schools, Scott et al. [35] found that a single-item measure can provide a reliable and valid assessment of youth physical activity relative to the existing Oxford Physical Activity Questionnaire (OPAQ) and accelerometry measures [35].

Screen time was measured using a question that asked respondents to report their daily average number of hours spent playing video games, watching TV, movies, or videos, texting, messaging, posting, or surfing the Internet during their leisure time over the past 7 days. This includes time on any screen, such as a TV, computer, tablet, smartphone, gaming device, or wearable technology. Participants who reported 2 h per day or less of screen time were considered compliant with the screen time recommendation [24]. Those who exceeded 2 h per day were deemed non-compliant with the screen time recommendation. Self-reported measures of screen time during leisure time have been indicated to have good psychometric properties among children and adolescents [36, 37]. For example, Schmitz et al. [37] found that the YRBSS weekday television viewing question, weekend television viewing, average television viewing, and computer use, had adequate reliability and validity for surveillance in a sample of 245 US middle school students.

Sleep duration was measured using a question that asked participants to indicate the average number of hours of sleep they get on school nights. Students who reported a sleep duration within the recommended ranges by age groups (9 to 11 h of sleep per night for those aged 11 to 13 years; 8 to 10 h of sleep per night for those aged 14 to 17 years, or 7 to 9 h of sleep per night for those aged 18 years or older) were considered compliant with the sleep duration recommendation. Those who reported a sleep duration outside the range for their age group were considered non-compliant with the sleep duration recommendation [24, 38]. In their systematic review of criterion validation of sleep time questionnaires for children and adolescents (considering accelerometers as the reference method), Nascimento-Ferreira et al. [39] found that self-reported items for sleep duration have good reliability and validity in this age group [39].

### Covariates

Covariates included in the analyses were age (measured in years), sex (females or males), ethnoracial background (including White, Black, East/Southeast Asian, South Asian, or other), subjective socioeconomic status (SES), and body mass index (BMI) z-score. Subjective SES was assessed using an adapted version of the MacArthur Scale for Subjective Social Status [40, 41]. The MacArthur Scale has been identified as a good measure of subjective social status [41]. BMI (kilograms/meters^2^) was calculated using self-reported body mass (kilograms) and height (meters), and after that, converted into z-scores based on the World Health Organization (WHO) reference data [42].

### Data analysis

All statistical analyses were conducted using Stata 16.0 (Stata Corporation, College Station, TX, USA). Taylor series linearization methods were employed to adjust for the cluster sampling design of the OSDUHS. Sex differences were examined by a design-adjusted Rao-Scott F-test statistic for categorical variables and an adjusted Wald test for continuous variables. Two-way interactions between compliance with the 24-h movement guidelines with consumption frequency of breakfast and fruits and vegetables were not significant. Univariable and multivariable ordered logistic regression models were used to examine the associations of compliance with the 24-h movement guidelines with consumption frequency of breakfast and fruits and vegetables. Models were adjusted for age, sex, ethnoracial background, subjective socioeconomic status, and BMI z-score. Odds ratios (OR) and 95% confidence intervals (CI) for both the unadjusted (Model 1) and adjusted (Model 2) models are presented.

## Results

Descriptive statistics provided in Table 1 indicate that more than half of the sample was females and 50% were of White ethnoracial background. Overall, 21% of participants met the physical activity recommendation, 24% met the screen time recommendation, and 33% met the sleep duration recommendation. Only 3.6% of participants met all three recommendations, whereas nearly 44.6% met none of the recommendations. Males were more compliant with the physical activity (p < 0.001), screen time (p < 0.05), and sleep duration (p < 0.001) recommendations than females. They were also more compliant with all three recommendations than females (p < 0.001). The median frequencies of breakfast consumption and fruit and vegetable intake were identical among females and males.

**Table 1:** Descriptive characteristics of participants

	Full sample (n = 12,759)	Females (n = 7,156)	Males (n = 5,603)	*p* value
Age (years)				
Mean (SD)	15.2 (1.8)	15.2 (1.9)	15.3 (1.7)	0.339
Ethnoracial background				
White	50.1	50.5	49.7	0.336
Black	8.8	9.3	8.4	
East/South-East Asian	14.7	13.7	15.7	
South Asian	10.0	10.0	10.0	
Other	16.4	16.6	16.2	
Subjective socioeconomic status				
Mean (SD)	6.9 (1.7)	6.9 (1.8)	6.9 (1.5)	< 0.001
Body mass index z-score				
Mean (SD)	0.3 (1.1)	0.3 (1.3)	0.3 (1.0)	0.578
Physical activity				
Not met	79.0	84.7	73.6	< 0.001
Met	21.0	15.3	26.5	
Screen time				
Not met	76.0	77.3	74.8	0.041
Met	24.0	22.7	25.2	
Sleep duration				
Not met	66.7	71.7	61.8	< 0.001
Met	33.3	28.3	38.2	
Combinations of movement recommendations				
Met none	44.6	50.3	39.1	< 0.001
Physical activity only	8.7	7.2	10.1	
Screen time only	9.9	11.4	8.6	
Sleep duration only	17.5	16.7	18.4	
Physical activity and screen time only	3.5	2.9	4.1	
Physical activity and sleep only	5.3	3.2	7.3	
Screen time and sleep duration only	7.0	6.3	7.6	
Met all three recommendations	3.6	2.2	5.0	
Breakfast consumption				
[Min: 1, Max: 4]				
Median	3	3	3	
Fruit and vegetable intake				
[Min: 1, Max: 7]				
Median	4	4	4	

Data are shown as weighted column %, unless otherwise indicated. *SD* standard deviation

Results from the univariable and multivariable ordered logistic regression models examining the association of compliance with the physical activity, screen time, and sleep duration recommendations with breakfast consumption and fruit and vegetable intake are summarized in Table 2. After adjustment for covariates, results indicate that compliance with all three recommendations was associated with more frequent breakfast consumption (OR: 2.77; 95% CI: 2.17–3.55) and fruit and vegetable intake (OR: 2.95; 95% CI: 2.41–3.62) compared to compliance with none of the recommendations. Compliance with the different combinations of recommendations was also associated with more frequent breakfast consumption and fruit and vegetable intake compared with none of the recommendations, with some exceptions. Compliance with the physical activity recommendation only or the screen time recommendation only was not associated with the frequency of breakfast consumption. Compliance with the screen time recommendation only was not associated with fruit and vegetable intake.

**Table 2:** Results of the ordered logistic regression analysis of the associations of compliance with different combinations of movement behaviour recommendations with breakfast consumption and fruit and vegetable intake among adolescents (N = 12,759)

	Breakfast consumption			Fruit and vegetable intake		
	OR	95% CI	*p* value	OR	95% CI	*p* value
Unadjusted						
Compliance with none	1			1		
Physical activity only	1.25	1.07–1.44	0.004	1.71	1.41–2.08	< 0.001
Screen time only	1.16	1.03–1.31	0.017	1.16	1.00–1.34	0.051
Sleep duration only	1.86	1.65–2.15	< 0.001	1.22	1.09–1.35	< 0.001
Physical activity and screen time only	1.83	1.41–2.36	< 0.001	2.24	1.75–2.86	< 0.001
Physical activity and sleep only	2.32	1.83–2.95	< 0.001	2.53	2.02–3.18	< 0.001
Screen time and sleep duration only	3.00	2.47–3.64	< 0.001	1.65	1.33–2.04	< 0.001
Met all three recommendations	3.70	2.88–4.74	< 0.001	3.50	2.87–4.25	< 0.001
Adjusted						
Compliance with none	1			1		
Physical activity only	1.09	0.93–1.27	0.280	1.67	1.36–2.04	< 0.001
Screen time only	1.12	0.99–1.26	0.073	1.09	0.95–1.26	0.224
Sleep duration only	1.76	1.54–2.01	< 0.001	1.17	1.04–1.30	0.007
Physical activity and screen time only	1.58	1.23– 2.03	< 0.001	2.05	1.59–2.64	< 0.001
Physical activity and sleep only	1.91	1.51–2.43	< 0.001	2.39	1.90–3.00	< 0.001
Screen time and sleep duration only	2.62	2.13–3.22	< 0.001	1.48	1.19–1.83	< 0.001
Met all three recommendations	2.77	2.17–3.55	< 0.001	2.95	2.41–3.62	< 0.001

*OR* odds ratio, *CI* confidence interval

Models are adjusted for age, sex, ethnoracial background, subjective socioeconomic status, and body mass index z-scores

Table 3 presents results of the univariable and multivariable ordered logistic regression models examining the association between the number of movement behaviour recommendations met with breakfast consumption and fruit and vegetable intake. After adjustment for covariates, results indicate that there was a dose–response relationship between the number of recommendations achieved by the students (3 > 2 > 1) and more frequent consumption of breakfast (p < 0.001) and fruits and vegetables (p < 0.001).

**Table 3:** Results of the ordered logistic regression analysis of the associations of number of movement behaviour recommendations met with breakfast consumption and fruit and vegetable intake among adolescents (N = 12,759)

	Breakfast consumption			Fruit and vegetable intake		
	OR	95% CI	*p* value	OR	95% CI	*p* value
Unadjusted						
None	1			1		
One	1.48	1.35–1.62	< 0.001	1.30	1.17–1.43	< 0.001
Two	2.46	2.09–2.89	< 0.001	2.02	1.73–2.37	< 0.001
Three	3.69	2.88–4.73	< 0.001	3.48	2.87–4.23	< 0.001
* p-*trend			< 0.001			< 0.001
Adjusted						
None	1			1		
One	1.37	1.25–1.51	< 0.001	1.24	1.12–1.37	< 0.001
Two	2.10	1.77–2.50	< 0.001	1.84	1.57–2.16	< 0.001
Three	2.77	2.17–3.55	< 0.001	2.92	2.38–3.57	< 0.001
* p-*trend			< 0.001			< 0.001

*OR* odds ratio, *CI* confidence interval

Covariates in the adjusted models included age, sex, ethnoracial background, subjective socioeconomic status, and body mass index z-scores

## Discussion

This study examined the associations of compliance with the physical activity, screen time, and sleep duration recommendations with frequency of breakfast consumption and fruit and vegetable intake in a province-wide representative sample of adolescents. Results showed that compliance with all three recommendations was associated with more frequent breakfast consumption and fruit and vegetable intake. There was a dose–response relationship between the number of recommendations achieved and more frequent consumption of breakfast and fruits and vegetables, with compliance with all three recommendations being the combination most strongly associated with the outcomes. These findings suggest that compliance with the physical activity, screen time, and sleep duration recommendations is a significant correlate of frequent breakfast consumption and fruit and vegetable intake among adolescents.

Many studies have investigated the relationships between physical activity, screen time, and sleep duration with eating behaviours including breakfast consumption and eating fruits and vegetables [17–23], though most of them considered movement behaviours individually and in isolation of each other, ignoring the intrinsic and empirical interactions between these co-dependent behaviours. There is only one study by Thivel et al. [25] we are aware of that has investigated dietary patterns in the context of compliance with the physical activity, screen time, and sleep duration recommendations. They found that compliance with the physical activity, screen time, and sleep duration recommendations was associated with better dietary patterns (composite scores) in a sample of more than 5800 children aged 9 to 11 years from 12 countries around the world. Thivel et al. used objective measures of physical activity and sleep duration, which provides more precise estimates of these movement behaviours. Nevertheless, our results provide evidence of associations between compliance with the physical activity, screen time, and sleep duration recommendations and specific eating behaviour indicators, including frequency of breakfast consumption and fruit and vegetable intake among adolescents. Research has identified adolescence as a nutritionally vulnerable time period [43], thus supporting the need for a better understanding of important lifestyle factors associated with eating behaviours in this age group.

Several mechanisms could explain the associations of physical activity, screen time, and sleep duration with unhealthy eating behaviours among adolescents. Physical inactivity and unhealthy eating behaviours are major contributors of excess weight and obesity in children and adolescents [44]. Regular physical activity may reduce levels of the body's stress hormones, such as adrenaline and cortisol [45] and improve mood [46–48], and subsequently decrease the chance of unhealthy eating behaviours. It also improves secretion of endorphins, which are chemicals in the brain that are the body's natural painkillers and mood elevators [49]. Regular physical activity also helps build confidence by improving body image, and thus reducing risk of unhealthy eating or disordered eating [48, 50]. Physical activity also generates a sense of accomplishment that boosts confidence and puts one’s mind in a more positive state that could favour making other healthy choices [51].

The link between screen time and unhealthy eating behaviours could be explained by several factors, including unhealthy eating behaviour as (1) a direct consequence of food advertising on electronic media platforms; (2) a concurrent activity to recreational screen time; (3) a consequence of body weight concerns; and (4) a result of displacement of available time for healthy eating. Food advertising plays an important role in food choices and preferences among youth. Research has shown that exposure to food advertisements on electronic media increases food intake in children and alters their eating choices and behaviours [52–54]. Evidence has also shown that excessive screen time is associated with more sweets and soft drinks consumption, and less frequent consumption of breakfast and fruits and vegetables [55–58]. Excessive screen time has been associated with body image concerns among adolescents [59–61], which in turn is associated with disordered eating [62]. Another possible explanation of the link between high screen time levels and unhealthy eating behaviours, such as skipping breakfast, is related to the displacement of other activities [63]. It is possible that excessive screen time directly decreases the amount of discretionary time available for eating breakfast.

Short sleep duration can lead to a dysregulation of the hypothalamic–pituitary–adrenal axis involving changes in cortisol secretion [64], which, in turn, increases stress [65] and affects eating behaviours. Research has shown that children and adolescents experiencing stress are more likely to adopt unhealthy eating habits, such as intake of high energy-dense (fatty) foods, snacking, skipping breakfast, and eating less fruits and vegetables [66–68]. It is also possible that short sleep offers more time and opportunities to eat unhealthy food, especially late at night when coupled with screen time activities. Evidence has shown that extending sleep duration among short sleepers facilitates appetite control and helps with controlling body weight [69].

Taken together, the mechanisms explaining the collective relations of all three movement behaviours with breakfast consumption and fruit and vegetable intake among adolescents could rely on different combinations of the aforementioned mechanisms. It is also possible that individuals who meet all three movement behaviour recommendations are more prone to make other healthy choices, as they might also be more likely to adopt healthier eating behaviours for optimal health benefits. Our findings suggest that health promotion approaches that address all three movement behaviours concurrently instead of single behaviours may bring about more health benefits as these behaviours are codependent and interact with one another [70].

Several limitations of this study should be considered. First, the cross-sectional nature of the data prevents inferences of causality between compliance with the movement behaviours and consumption frequency of breakfast and fruits and vegetables. Future research using a longitudinal design is needed to better ascertain directionality of findings. Second, the data are self-reported and may therefore be affected by recall and desirability biases. Despite these limitations of self-reported measures of movement behaviours, they have been indicated to have good validity in comparison with objective tools in children and adolescents [35–37, 39]. Third, there is a possibility of residual confounding by unmeasured variables (e.g., dieting, being an athlete, medication use, chronic conditions), which is always a possibility in epidemiology. Fourth, the response rate in the 2019 OSDUHS was relatively low at 59%, which could have induced nonresponse bias. However, a comparison of selected characteristics in classes in which the class participation rate was low with those in which the rate was higher suggested no compelling evidence that the nonparticipation rate produced appreciable bias [26]. Another limitation is related to the failure to account for any family-related confounders in the analysis. Although adolescents do gain a certain level of autonomy, they are still influenced by parental decisions, values, and practices. Future research is therefore needed to adjust for possible effect of family-related confounders. Finally, this study includes students in grades 7 to 12 within the publicly funded school system in Ontario and is not representative of all students in Canada.

## Conclusion

Compliance with the physical activity, screen time, and sleep duration recommendations was associated with more frequent consumption of breakfast and fruits and vegetables in a representative sample of adolescents in Ontario, Canada. Compliance with all three movement behaviour recommendations was more strongly associated with more frequent consumption of breakfast and fruits and vegetables, while compliance with two guideline recommendations being better than compliance with one, and compliance with one being better than compliance with none. These findings provide further support to the 24-h movement guidelines for general health. Ongoing intervention efforts and messages should encourage adolescents to increase their engagement in MVPA, reduce their recreational screen time, and get a sufficient amount of sleep each day. Further research using prospective data is needed to confirm causality.

## Data Availability

Due to institutional restrictions data cannot be made available in the manuscript, the supplemental files, or a public repository. Data are available to all researchers after an institutional application is approved. To access data contact osduhs@camh.ca for further instructions.

## References

[1] World Health Organization. Adolescent development. In: Maternal, newborn, child and adolescent health, World Health Organization, Geneva, Switzerland, 2020.

[2] Marcia JE. Identity in adolescence. Handb Adolesc Psychol. 1980;9(11):159–87. 10.1007/BF02087928 24318013

[3] Soliman A, De Sanctis V, Elalaily R. Nutrition and pubertal development. Indian J Endocrinol Metab. 2014;18(Suppl 1):S39–47. 10.4103/2230-8210.145073. 25538876 10.4103/2230-8210.145073 PMC4266867

[4] Sampasa-Kanyinga H, Roumeliotis P, Farrow CV, Shi YF. Breakfast skipping is associated with cyberbullying and school bullying victimization. A school-based cross-sectional study. Appetite. 2014;79:76–82. 10.1016/j.appet.2014.04.007. 24746660 10.1016/j.appet.2014.04.007

[5] Mullie P, Clarys P, Ridder D, Deriemaeker P, Duvigneaud N, Hebbelinck M, et al. Breakfast frequency and fruit and vegetable consumption in Belgian adolescents. A cross-sectional study. Nutr Food Sci. 2006;36:315–26. 10.1108/00346650610703162.

[6] Shaw ME. Adolescent breakfast skipping: an Australian study. Adolescence. 1998;33(132):851–61. 9886013

[7] Mullan B, Wong C, Kothe E, O’Moore K, Pickles K, Sainsbury K. An examination of the demographic predictors of adolescent breakfast consumption, content, and context. BMC Public Health. 2014;14:264. 10.1186/1471-2458-14-264. 24645936 10.1186/1471-2458-14-264 PMC4000053

[8] Adolphus K, Lawton CL, Dye L. The effects of breakfast on behavior and academic performance in children and adolescents. Front Hum Neurosci. 2013;7:425. 10.3389/fnhum.2013.00425. 23964220 10.3389/fnhum.2013.00425 PMC3737458

[9] Rampersaud GC, Pereira MA, Girard BL, Adams J, Metzl JD. Breakfast habits, nutritional status, body weight, and academic performance in children and adolescents. J Am Diet Assoc. 2005;105(5):743–60; quiz 61–2. 10.1016/j.jada.2005.02.007. 15883552 10.1016/j.jada.2005.02.007

[10] American Academy of Pediatrics Healthy Children. Breakfast for learning, 2019. https://www.healthychildren.org/English/healthy-living/nutrition/Pages/Breakfast-for-Learning.aspx. Accessed 14 July 2021 2021.

[11] Slavin JL, Lloyd B. Health benefits of fruits and vegetables. Adv Nutr. 2012;3(4):506–16. 10.3945/an.112.002154. 22797986 10.3945/an.112.002154 PMC3649719

[12] Liu RH. Health-promoting components of fruits and vegetables in the diet. Adv Nutr. 2013;4(3):384S-S392. 10.3945/an.112.003517. 23674808 10.3945/an.112.003517 PMC3650511

[13] Willett WC, Sacks F, Trichopoulou A, Drescher G, Ferro-Luzzi A, Helsing E, et al. Mediterranean diet pyramid: a cultural model for healthy eating. Am J Clin Nutr. 1995;61(6 Suppl):1402S-S1406. 10.1093/ajcn/61.6.1402S. 7754995 10.1093/ajcn/61.6.1402S

[14] Adelantado-Renau M, Beltran-Valls MR, Esteban-Cornejo I, Martinez-Vizcaino V, Santaliestra-Pasias AM, Moliner-Urdiales D. The influence of adherence to the Mediterranean diet on academic performance is mediated by sleep quality in adolescents. Acta Paediatr. 2019;108(2):339–46. 10.1111/apa.14472. 30019348 10.1111/apa.14472

[15] Esteban-Cornejo I, Izquierdo-Gomez R, Gomez-Martinez S, Padilla-Moledo C, Castro-Pinero J, Marcos A, et al. Adherence to the Mediterranean diet and academic performance in youth: the UP&DOWN study. Eur J Nutr. 2016;55(3):1133–40. 10.1007/s00394-015-0927-9. 25975266 10.1007/s00394-015-0927-9

[16] Tapia-Serrano MA, Esteban-Cornejo I, Rodriguez-Ayllon M, Vaquero-Solís M, Sánchez-Oliva D, Sánchez-Miguel PA. Adherence to the Mediterranean diet and academic performance in adolescents: does BMI status moderate this association? Clin Nutr. 2021;40(6):4465–72. 10.1016/j.clnu.2020.12.036. 33495088 10.1016/j.clnu.2020.12.036

[17] Arora M, Nazar GP, Gupta VK, Perry CL, Reddy KS, Stigler MH. Association of breakfast intake with obesity, dietary and physical activity behavior among urban school-aged adolescents in Delhi, India: results of a cross-sectional study. BMC Public Health. 2012;12(1):1–12. 23075030 10.1186/1471-2458-12-881 PMC3549919

[18] Sandercock G, Voss C, Dye L. Associations between habitual school-day breakfast consumption, body mass index, physical activity and cardiorespiratory fitness in English schoolchildren. Eur J Clin Nutr. 2010;64(10):1086–92. 20683459 10.1038/ejcn.2010.145

[19] Chaput JP. Sleep patterns, diet quality and energy balance. Physiol Behav. 2014;134:86–91. 10.1016/j.physbeh.2013.09.006. 24051052 10.1016/j.physbeh.2013.09.006

[20] Zakrzewski-Fruer JK, Gillison FB, Katzmarzyk PT, Mire EF, Broyles ST, Champagne CM, et al. Association between breakfast frequency and physical activity and sedentary time: a cross-sectional study in children from 12 countries. BMC Public Health. 2019;19(1):222. 10.1186/s12889-019-6542-6. 30791951 10.1186/s12889-019-6542-6 PMC6385453

[21] Silva DAS, Silva RJdS. Association between physical activity level and consumption of fruit and vegetables among adolescents in northeast Brazil. Rev Paul Pediatr. 2015;33:167–73. 25887930 10.1016/j.rpped.2014.09.003 PMC4516370

[22] Pearson N, Biddle SJ. Sedentary behavior and dietary intake in children, adolescents, and adults. A systematic review. Am J Prev Med. 2011;41(2):178–88. 10.1016/j.amepre.2011.05.002. 21767726 10.1016/j.amepre.2011.05.002

[23] Bel S, Michels N, De Vriendt T, Patterson E, Cuenca-García M, Diethelm K, et al. Association between self-reported sleep duration and dietary quality in European adolescents. Br J Nutr. 2013;110(5):949–59. 10.1017/s0007114512006046. 23506795 10.1017/S0007114512006046

[24] Tremblay MS, Carson V, Chaput JP, Connor Gorber S, Dinh T, Duggan M, et al. Canadian 24-Hour movement guidelines for children and youth: an integration of physical activity, sedentary behaviour, and sleep. Appl Physiol Nutr Metab. 2016;41(6 Suppl 3):S311–27. 10.1139/apnm-2016-0151. 27306437 10.1139/apnm-2016-0151

[25] Thivel D, Tremblay MS, Katzmarzyk PT, Fogelholm M, Hu G, Maher C, et al. Associations between meeting combinations of 24-hour movement recommendations and dietary patterns of children: a 12-country study. Prev Med. 2019;118:159–65. 10.1016/j.ypmed.2018.10.025. 30393016 10.1016/j.ypmed.2018.10.025

[26] Boak A, Elton-Marshall T, Mann RE, Hamilton HA. Drug use among Ontario students, 1977–2019: Detailed findings from the Ontario Student Drug Use and Health Survey (OSDUHS). Toronto, ON: Centre for Addiction and Mental Health. 2020.

[27] Courser MW, Shamblen SR, Lavrakas PJ, Collins D, Ditterline P. The impact of active consent procedures on nonresponse and nonresponse error in youth survey data: evidence from a new experiment. Eval Rev. 2009;33(4):370–95. 10.1177/0193841X09337228. 19506295 10.1177/0193841X09337228 PMC2705468

[28] Kann L, McManus T, Harris WA, Shanklin SL, Flint KH, Queen B, et al. Youth risk behavior surveillance—United States, 2017. MMWR Surveill Summ. 2018;67(8):1. 10.15585/mmwr.ss6708a1 PMC6002027 29902162

[29] Currie C, Nic Gabhainn S, Godeau E. The Health Behaviour in School-aged Children: WHO Collaborative Cross-National (HBSC) study: origins, concept, history and development 1982–2008. Int J Public Health. 2009;54(Suppl 2):131–9. 10.1007/s00038-009-5404-x. 19639260 10.1007/s00038-009-5404-x

[30] Bourdeaudhuij I, Klepp K-I, Due P, Perez-Rodrigo C, de Almeida M, Wind M, et al. Reliability and validity of a questionnaire to measure personal, social and environmental correlates of fruit and vegetable intake in 10–11-year-old children in five European countries. Public Health Nutr. 2005;8:189–200. 10.1079/PHN2004673. 15877912 10.1079/phn2004673

[31] Zullig KJ, Pun S, Patton JM, Ubbes VA. Reliability of the 2005 middle school youth risk behavior survey. J Adolesc Health. 2006;39(6):856–60. 17116516 10.1016/j.jadohealth.2006.07.008

[32] Brener N, Collins J, Kann L, Warren C, Williams B. Reliability of the youth risk behavior survey questionnaire. Am J Epidemiol. 1995;141(6):575–80. 7900725 10.1093/oxfordjournals.aje.a117473

[33] Vereecken C, Dupuy M, Rasmussen M, Kelly C, Nansel TR, Al Sabbah H, et al. Breakfast consumption and its socio-demographic and lifestyle correlates in schoolchildren in 41 countries participating in the HBSC study. Int J Public Health. 2009;54(Suppl 2):180–90. 10.1007/s00038-009-5409-5. 19639257 10.1007/s00038-009-5409-5 PMC3408388

[34] Eaton DK, Olsen EOM, Brener ND, Scanlon KS, Kim SA, Demissie Z, et al. A comparison of fruit and vegetable intake estimates from three survey question sets to estimates from 24-hour dietary recall interviews. J Acad Nutr Diet. 2013;113(9):1165–74. 10.1016/j.jand.2013.05.013. 23871104 10.1016/j.jand.2013.05.013 PMC4655105

[35] Scott JJ, Morgan PJ, Plotnikoff RC, Lubans DR. Reliability and validity of a single-item physical activity measure for adolescents. J Paediatr Child Health. 2015;51(8):787–93. 25643749 10.1111/jpc.12836

[36] Lubans DR, Hesketh K, Cliff DP, Barnett LM, Salmon J, Dollman J, et al. A systematic review of the validity and reliability of sedentary behaviour measures used with children and adolescents. Obes Rev. 2011;12(10):781–99. 10.1111/j.1467-789X.2011.00896.x. 21676153 10.1111/j.1467-789X.2011.00896.x

[37] Schmitz KH, Harnack L, Fulton JE, Jacobs DR Jr, Gao S, Lytle LA, et al. Reliability and validity of a brief questionnaire to assess television viewing and computer use by middle school children. J Sch Health. 2004;74(9):370–7. 10.1111/j.1746-1561.2004.tb06632.x. 15656264 10.1111/j.1746-1561.2004.tb06632.x

[38] Hirshkowitz M, Whiton K, Albert SM, Alessi C, Bruni O, DonCarlos L, et al. National Sleep Foundation’s sleep time duration recommendations: methodology and results summary. Sleep Health. 2015;1(1):40–3. 10.1016/j.sleh.2014.12.010. 29073412 10.1016/j.sleh.2014.12.010

[39] Nascimento-Ferreira MV, Collese TS, de Moraes ACF, Rendo-Urteaga T, Moreno LA, Carvalho HB. Validity and reliability of sleep time questionnaires in children and adolescents: a systematic review and meta-analysis. Sleep Med Rev. 2016;30:85–96. 26921735 10.1016/j.smrv.2015.11.006

[40] Goodman E, Huang B, Schafer-Kalkhoff T, Adler NE. Perceived socioeconomic status: a new type of identity that influences adolescents’ self-rated health. J Adolesc Health. 2007;41(5):479–87. 10.1016/j.jadohealth.2007.05.020. 17950168 10.1016/j.jadohealth.2007.05.020 PMC2204090

[41] Goodman E, Adler NE, Kawachi I, Frazier AL, Huang B, Colditz GA. Adolescents’ perceptions of social status: development and evaluation of a new indicator. Pediatrics. 2001;108(2):E31. 10.1542/peds.108.2.e31. 11483841 10.1542/peds.108.2.e31

[42] World Health Organization. WHO Anthro (version 3.2.2) and macros. World Health Organization, Geneva, Switzerland. 2011.

[43] Lifshitz F, Tarim O, Smith MM. Nutrition in adolescence. Endocrinol Metab Clin North Am. 1993;22(3):673–83. 8243454

[44] Sahoo K, Sahoo B, Choudhury AK, Sofi NY, Kumar R, Bhadoria AS. Childhood obesity: causes and consequences. J Fam Med Prim Care. 2015;4(2):187–92. 10.4103/2249-4863.154628. 10.4103/2249-4863.154628 PMC4408699 25949965

[45] Stults-Kolehmainen MA, Sinha R. The effects of stress on physical activity and exercise. Sports Med. 2014;44(1):81–121. 10.1007/s40279-013-0090-5. 24030837 10.1007/s40279-013-0090-5 PMC3894304

[46] Biddle SJ, Asare M. Physical activity and mental health in children and adolescents: a review of reviews. Br J Sports Med. 2011;45(11):886–95. 10.1136/bjsports-2011-090185. 21807669 10.1136/bjsports-2011-090185

[47] Karen JC, Wendell CT. Effects of physical activity on psychological variables in adolescents. Pediatr Exerc Sci. 1994;6(4):406–23. 10.1123/pes.6.4.40610.1123/pes.6.4.40610.1123/pes.6.4.40610.1123/pes.6.4.406.

[48] Ekeland E, Heian F, Hagen KB, Abbott J, Nordheim L. Exercise to improve self-esteem in children and young people. Cochrane Database Syst Rev. 2004;1: Cd003683. 10.1002/14651858.CD003683.pub2. 10.1002/14651858.CD003683.pub2 PMC12935395 14974029

[49] Jain A, Mishra A, Shakkarpude J, Lakhani P. Beta endorphins: the natural opioids. Int J Chem Stud. 2019;7:323–32.

[50] Zamani Sani SH, Fathirezaie Z, Brand S, Pühse U, Holsboer-Trachsler E, Gerber M, et al. Physical activity and self-esteem: testing direct and indirect relationships associated with psychological and physical mechanisms. Neuropsychiatr Dis Treat. 2016;12:2617–25. 10.2147/NDT.S116811. 27789950 10.2147/NDT.S116811 PMC5068479

[51] Fox KR. The influence of physical activity on mental well-being. Public Health Nutr. 1999;2(3a):411–8. 10.1017/S1368980099000567. 10610081 10.1017/s1368980099000567

[52] Boyland EJ, Harrold JA, Kirkham TC, Corker C, Cuddy J, Evans D, et al. Food commercials increase preference for energy-dense foods, particularly in children who watch more television. Pediatrics. 2011;128(1):e93-100. 10.1542/peds.2010-1859. 21708808 10.1542/peds.2010-1859

[53] Andreyeva T, Kelly IR, Harris JL. Exposure to food advertising on television: associations with children’s fast food and soft drink consumption and obesity. Econ Hum Biol. 2011;9(3):221–33. 10.1016/j.ehb.2011.02.004. 21439918 10.1016/j.ehb.2011.02.004

[54] Harris JL, Bargh JA, Brownell KD. Priming effects of television food advertising on eating behavior. Health Psychol. 2009;28(4):404–13. 10.1037/a0014399. 19594263 10.1037/a0014399 PMC2743554

[55] Santaliestra-Pasias AM, Mouratidou T, Verbestel V, Huybrechts I, Gottrand F, Le Donne C, et al. Food consumption and screen-based sedentary behaviors in European adolescents: the HELENA study. Arch Pediatr Adolesc Med. 2012;166(11):1010–20. 10.1001/archpediatrics.2012.646. 22945250 10.1001/archpediatrics.2012.646

[56] Vereecken CA, Todd J, Roberts C, Mulvihill C, Maes L. Television viewing behaviour and associations with food habits in different countries. Public Health Nutr. 2006;9(2):244–50. 16571179 10.1079/phn2005847

[57] Sampasa-Kanyinga H, Chaput JP. Consumption of sugar-sweetened beverages and energy drinks and adherence to physical activity and screen time recommendations among adolescents. Int J Adolesc Med Health. 2016. 10.1515/ijamh-2015-0098. 26926857 10.1515/ijamh-2015-0098

[58] Sampasa-Kanyinga H, Chaput JP, Hamilton HA. Associations between the use of social networking sites and unhealthy eating behaviours and excess body weight in adolescents. Br J Nutr. 2015;114(11):1941–7. 10.1017/S0007114515003566. 26400488 10.1017/S0007114515003566

[59] Tiggemann M, Slater A. NetGirls: the Internet, Facebook, and body image concern in adolescent girls. Int J Eat Disord. 2013;46(6):630–3. 10.1002/eat.22141. 23712456 10.1002/eat.22141

[60] Tiggemann M, Slater A. NetTweens: the Internet and body image concerns in preteenage girls. J Early Adolesc. 2014;34(5):606–20.

[61] Meier EP, Gray J. Facebook photo activity associated with body image disturbance in adolescent girls. Cyberpsychol Behav Soc Netw. 2014;17(4):199–206. 10.1089/cyber.2013.0305. 24237288 10.1089/cyber.2013.0305

[62] Striegel-Moore RH, Cachelin FM. Body image concerns and disordered eating in adolescent girls: risk and protective factors. In: Johnson NG, Roberts MC, Worell J, editors. Beyond appearance: a new look at adolescent girls. Washington: American Psychological Association; 1999. p. 85–108.

[63] Pearson N, Biddle SJH. Sedentary behavior and dietary intake in children, adolescents, and adults: a systematic review. Am J Prev Med. 2011;41(2):178–88. 10.1016/j.amepre.2011.05.002. 21767726 10.1016/j.amepre.2011.05.002

[64] Romeo RD. The teenage brain: the stress response and the adolescent brain. Curr Dir Psychol Sci. 2013;22(2):140–5. 10.1177/0963721413475445. 25541572 10.1177/0963721413475445 PMC4274618

[65] Fredriksen K, Rhodes J, Reddy R, Way N. Sleepless in Chicago: tracking the effects of adolescent sleep loss during the middle school years. Child Dev. 2004;75(1):84–95. 15015676 10.1111/j.1467-8624.2004.00655.x

[66] Yau YHC, Potenza MN. Stress and eating behaviors. Minerva Endocrinol. 2013;38(3):255–67. 24126546 PMC4214609

[67] Tajik E, Latiffah AL, Awang H, Siti Nur’Asyura A, Chin YS, Azrin Shah AB, et al. Unhealthy diet practice and symptoms of stress and depression among adolescents in Pasir Gudang, Malaysia. Obes Res Clin Pract. 2016;10(2):114–23. 10.1016/j.orcp.2015.06.001. 26204813 10.1016/j.orcp.2015.06.001

[68] Cartwright M, Wardle J, Steggles N, Simon A, Croker H, Jarvis M. Stress and dietary practices in adolescents. Health psychol. 2003;22:362–9. 10.1037/0278-6133.22.4.362. 12940392 10.1037/0278-6133.22.4.362

[69] Chaput J-P, Dutil C. Lack of sleep as a contributor to obesity in adolescents: impacts on eating and activity behaviors. Int J Behav Nutr Phys Act. 2016;13(1):1–9. 27669980 10.1186/s12966-016-0428-0 PMC5037605

[70] Dumuid D, Olds T, Sawyer SM. Moving beyond more: towards a healthy balance of daily behaviours. Lancet. 2021;398(10298):373–4. 10.1016/s0140-6736(21)01600-7. 34302761 10.1016/S0140-6736(21)01600-7

